# Model-based risk assessment of dengue fever transmission in Xiamen City, China

**DOI:** 10.3389/fpubh.2023.1079877

**Published:** 2023-02-13

**Authors:** Zhinan Guo, Weikang Liu, Xingchun Liu, Buasiyamu Abudunaibi, Li Luo, Sihan Wu, Bin Deng, Tianlong Yang, Jiefeng Huang, Shenggen Wu, Lei Lei, Zeyu Zhao, Zhuoyang Li, Peihua Li, Chan Liu, Meirong Zhan, Tianmu Chen

**Affiliations:** ^1^Xiamen Center for Disease Control and Prevention, Xiamen, Fujian, China; ^2^State Key Laboratory of Molecular Vaccinology and Molecular Diagnostics, School of Public Health, Xiamen University, Xiamen, Fujian, China; ^3^Fujian Provincial Center for Disease Control and Prevention, Fuzhou, Fujian, China

**Keywords:** dengue fever, mathematical model, risk assessment, vector investigation, insecticide resistance monitoring

## Abstract

**Background:**

Quantitative assessment of the risk of local transmission from imported dengue cases makes a great challenge to the development of public health in China. The purpose of this study is to observe the risk of mosquito-borne transmission in Xiamen City through ecological and insecticide resistance monitoring. Quantitative evaluation of mosquito insecticide resistance, community population and the number of imported cases affecting the transmission of dengue fever (DF) in Xiamen was carried out based on transmission dynamics model, so as to reveal the correlation between key risk factors and DF transmission.

**Methods:**

Based on the dynamics model and combined with the epidemiological characteristics of DF in Xiamen City, a transmission dynamics model was built to simulate the secondary cases caused by imported cases to evaluate the transmission risk of DF, and to explore the influence of mosquito insecticide resistance, community population and imported cases on the epidemic situation of DF in Xiamen City.

**Results:**

For the transmission model of DF, when the community population is between 10,000 and 25,000, changing the number of imported DF cases and the mortality rate of mosquitoes will have an impact on the spread of indigenous DF cases, however, changing the birth rate of mosquitoes did not gain more effect on the spread of local DF transmission.

**Conclusions:**

Through the quantitative evaluation of the model, this study determined that the mosquito resistance index has an important influence on the local transmission of dengue fever caused by imported cases in Xiamen, and the Brayton index can also affect the local transmission of the disease.

## Introduction

Dengue fever (DF) is a viral infectious disease transmitted by *Aedes albopictus* and *Aedes aegypti*. There are four types of serotype for DF virus, namely DENV-1, DENV-2, DENV-3, and DENV-4 (1, 2). People infected with dengue virus can have fever, dengue hemorrhagic fever, and other clinical symptoms. For severe cases, they suffer from dengue shock syndrome and death (3, 4). One recent research indicates that there are 390 million (95% credible interval 284–528 million) new DF cases per year in the world, of which about 12,000 death (1, 5). More researches have estimated that the incidence of DF as increased nearly 30 times compared with 50 years ago, and about one third of the world's population are at risk of DF infection (6–8). At the same time, due to the lack of specific treatment and effective vaccination against DF, the epidemic of DF has become one of the major global public health problem, causing serious disease burden to many countries and regions (2).

In recent years, the annual reported incidence of DF in China has shown an obvious upward trend (9). Besides Guangdong, Guangxi, Hainan, and other areas, which are with high incidence of DF, the central and northern regions of China also have reported cases of DF (10, 11). Xiamen City, as a special economic zone in China approved by the State Council, has frequent personnel exchanges with countries and regions with high incidence of DF cases in Southeast Asia. Therefore, Xiamen City is facing a grim situation of prevention and control of imported DF cases. Meanwhile, located in the subtropical maritime monsoon climate zone, Xiamen City has suitable temperature and humidity for mosquito-borne transmission. Thus, when dengue virus is imported in the right season, the risk of disease transmission was higher. Consequently, the epidemic prevention departments need to quantitatively understand the potential risk factors of DF transmission, accordingly, promote the formulation of more effective control strategies and early warning surveillance.

Prior to this study, there have been many researches on DF by using mathematical models (12–14). A study established a human-vector coupled dynamic model to evaluate the effect of intervention measures taken in DF outbreak (12). In another study, the generalized mixed linear equation was introduced into the SIR dynamics model, which emphasized the influence of meteorological conditions on *Ae. albopictus* and discussed the influence of meteorological factors on DF propagation (13). In the meantime, the researchers, *via* time series regression tree analysis, have found that the timeliness of DF monitoring system in DF transmission in Zhongshan City, China and meteorological factors have influenced the local DF incidence (14). Nevertheless, in the research of potential factors affecting DF communication, there is no quantitative evaluation of DF potential communication risk factors based on communication dynamics model in China.

In this paper, we developed a Hosts-Vectors Susceptible–Exposed–Infectious–Asymptomatic–Recovered (SEIAR) transmission dynamics model to quantitatively evaluate the influence of mosquito insecticide resistance and imported cases on DF transmission in Xiamen City. In addition, through SEIAR model, it will help guiding public health departments in Xiamen City and other areas to put forward scientific strategies and early warning and forecasting systems for controlling DF transmission.

## Materials and methods

### Study area

Xiamen (118°04′04″E, 24°26′46″N), located in the southeast of China, is a coastal city with a population of 4.29 million. It is an important city in Fujian Province and occupies an area of 1,700.61 square kilometers. The city has six districts under its jurisdiction, including 26 streets, 12 towns, 361 communities, and 147 villages. The city belongs to is subtropical monsoon climate with a yearly average temperature of 21°C and an average annual rainfall of about 1,315 mm, most of which is mainly concentrated from May to August. According to the dengue surveillance data from 2005 to 2019 in Xiamen City, *Ae. Albopictus* was the only vector species in the city, and imported DF cases were the majority cases (15).

### Case definition and case-finding

In the study, an indigenous case was defined as an individual who are infected with DF and have not left this city (current address) within 14 days before the onset of disease. An imported DF cases was defined as an infected patient who had been to DF epidemic regions within 14 days after the onset of disease.

All the DF cases were identified following the diagnostic criteria announced by National Health Commission of the People's Republic of China (WS216-2008) (16):

- Suspected case: an individual who had been to DF epidemic regions within 14 days or there has been a DF case around his/her residence or workplace (within a radius of 200 m) within 1 month, along with having one or more symptoms, and no specific diagnosis has been confirmed as other diseases.
- Clinically diagnosed case: a suspected case with leucopenia or thrombocytopenia, or a suspected case whose serum specific immunoglobulin IgG or IgM test is positive.
- Laboratory-confirmed case: a clinically diagnose case has one or more of the following test results:
  - Serum tested positive for DENV RNA by real-time PCR;
  - An IgG titer in the recovery period is 4-fold higher than that in the acute period;
  - Isolation of the DENV from the blood, tissue or cerebrospinal fluid of a patient with acute infection.

### Vector investigation and insecticide resistance monitoring

In order to obtain the relevant parameters in the model, we have assessed the density of *Ae. albopictus* in Xiamen City by three ecological monitoring methods: Brayton index method, Container index method and Human-baited double net trapping method. And mosquito surveillance was performed twice a month in two districts (Huli and Xiang'an) of Xiamen City from May to November in 2020.

#### Brayton index

An investigation of BI was conducted from May 1st to November 1st in Xiamen City. In four residential areas of different geographical locations, there were no <100 households selected in each monitoring district. For other habitats, such as park, bamboo forests, old tire dumps, waste collecting stations, 50 households need to be collected. Then we recorded the occurrence of *Ae. albopictus* larvae in all indoor and outdoor water containers (17). For identifying the species, we collected the larvae and brought them back to Center for Disease Control and Prevention (CDC) laboratory for breeding to adult mosquitoes and thereby making the identification (Supplementary Text S1, p. 1). Finally, we use the following formula to calculate the BI:

$$\begin{array}{l} \text{BI}=\frac{\text{Number of positive containers of }Ae.albopictus}{\text{Number of households surveyed}}\times 100\% \end{array}$$

#### Container index

The site selected for monitoring Container index is consistent with the above-mentioned site, and should be used to monitoring the CI first. In each monitoring district, it has to be no <100 containers in four residential areas in different geographical locations for 4 days, and the distance between each container is 25–30 m. On the fourth day, the adult mosquitoes were monitored, and the species were identified after larvae grow up (Supplementary Text S1, p. 1, 2). We used the following formula to calculate the CI:

$$\begin{array}{l} \text{CI}=\frac{\text{Number of positive containers }}{\text{Number of effective containers}}\times 100\text{\%} \end{array}$$

#### Human-baited double net trapping

Adult mosquito monitoring mainly adopts the method of human-baited double net trapping. We have selected four different habitats, each with two nets more than 100 meters apart. In the afternoon (15:00–18:00), when the vector activity was at its peak, the attractor had both legs exposed in an internal closed mosquito net, and the collector was in trousers (18). Mosquito repellent was not used during the monitoring process. An electric mosquito absorber was used to quickly collect vector Aedes that fell on the mosquito net, then then leave as soon as possible. The monitoring lasted for 30 min and the vector Aedes were collected from each mosquito net (Supplementary Text S1, p. 2). We use the formula to calculate the inducement index:

$$\begin{array}{l} \text{HDN}=\frac{\text{Number of female mosquitoes captured }}{\text{Number of mosquito nets}\times 30\text{ minutes}}\\ \times \;60\text{ minutes}/\text{hours} \end{array}$$

### Insecticide resistance monitoring

It was mainly the end of 3rd instar to the beginning of 4th instar larvae that were chosen to monitor the insecticide resistance. And we adopted the impregnation technique recommended by WHO (refer to GB/T26347-2010) to determine LC50 and calculate RR (17). In the meantime, the WHO recommended contact tube method (refer to GB/T26347-2010) was used to monitor the resistance of adult mosquitoes to insecticides, and thereby the mortality of adult mosquitoes at diagnostic dose was determined. We selected 11 different types of pesticides, namely: 0.2% bendiocarb, 0.2% fenitrothion, 0.03% deltamethrin, 0.04% permethrin, 0.5% propoxur, 0.5% malathion, 0.08% beta-cypermethrin, 0.07% lambda-cyhalothrin, 2% chlorpyrifos, 0.4% beta-cypermethrin. See Supplementary Text S1, p. 3–6 for specific experimental methods.

### Dynamic model of DF transmission

**Figure 1:**
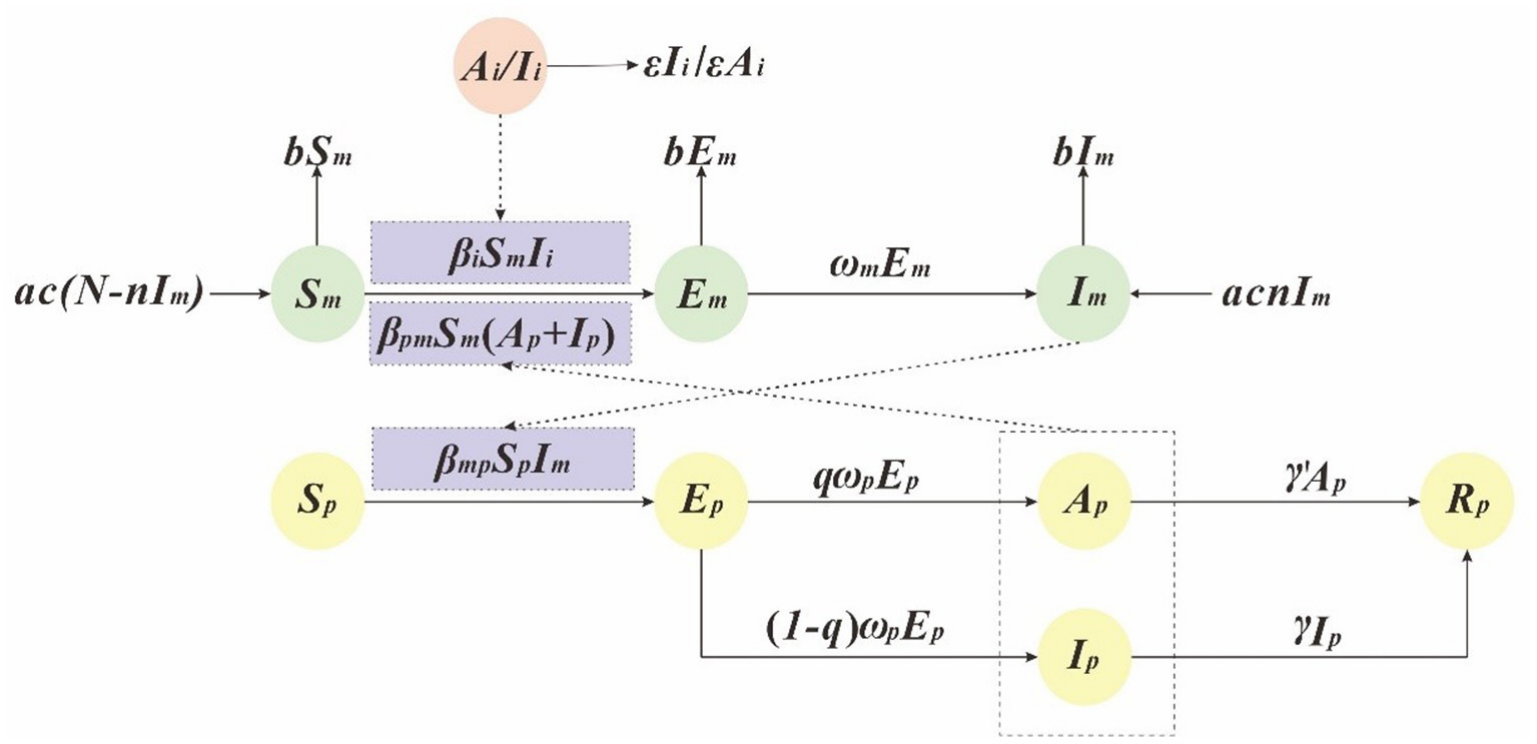
The flowchart of the development of the dengue fever transmission model.

In this study, we has built a dynamics model based on SEIAR to simulate the transmission of the dengue virus (9, 12, 19). And in this model, people were divided into the following five compartments: susceptible (*S*_*p*_), exposed (*E*_*p*_), infectious (*I*_*p*_), asymptomatic (*A*_*p*_), removed (*R*_*p*_). *A*_*i*_/*I*_*i*_ refers to imported DF cases. Vectors were divided into the following three compartments: susceptible (*S*_*m*_), exposed (*E*_*m*_), infectious (*I*_*m*_), see Table 1. And the interaction between the human and vector is presented in Figure 1.

**Table 1:** Variables in the SEIAR-SEI model.

**Variables**	**Description**	**Unit**
*S_*p*_*	Susceptible individuals	Individuals
*E_*p*_*	Exposed individuals	Individuals
*I_*p*_*	Infectious individuals	Individuals
*A_*p*_*	Asymptomatic individuals	Individuals
*R_*p*_*	Recovered/removed individuals	Individuals
*S_*m*_*	Susceptible vectors	Individuals
*E_*m*_*	Exposed vectors	Individuals
*I_*m*_*	Infectious vectors	Individuals
*A_*i*_/I_*i*_*	Imported infectious/asymptomatic individuals	Individuals

This model is based on the following assumptions:

1. The model assumes that the propagation coefficient of *S*_*p*_ and *I*_*m*_ after effective propagation is β_*mp*_, the transmission rate from *A*_*i*_/*I*_*i*_ to *S*_*m*_ is β_*i*_, and the transmission rate from *S*_*m*_ to *I*_*p*_ and *A*_*p*_ is β_*pm*_. Therefore, at time *t*, the number of newly infected DF is β_*pm*_*S*_*p*_*I*_*m*_, and the number of newly infected vectors is β_*i*_*S*_*m*_*I*_*i*_ + β_*pm*_*S*_*m*_ *(A*_*p*_ + *I*_*p*_*)*.
2. The model assumes that the proportion of recessive infection is q and the latency is $\frac{1}{\omega }$, So at time *t*, the number of individual who changed from *E*_*p*_ to *A*_*p*_ and *I*_*p*_ were *q*ω_*p*_*E*_*p*_ and (1–*q*)ω_*p*_*E*_*p*_.
3. The model assumes that the time interval from onset to first diagnosis of case *I*_*p*_ was $\frac{1}{\gamma }$, so at time *t*, the number of people who changed from *I*_*p*_ to *R*_*p*_ is γ*I*_*p*_. In addition, we assume that the infection period of asymptomatic infected person A is $\frac{1}{\gamma^{\prime }}$, so at time *t*, the number of people who changed from A to R is γ′*A*_*p*_.
4. The model assumes that the natural vectors mortality rate is *b*, the natural birth rate is *a*, and the vertical transmission ratio of dengue virus by mosquitoes is *n*.

The mathematical model is described by the following ordinary differential equations (ODE):

$$\begin{array}{l} \frac{dS_{p}}{dt}=-\beta_{mp}S_{p}I_{m}\\ \frac{dE_{p}}{dt}=\beta_{mp}S_{p}I_{m}-\omega_{p}E_{p}\\ \frac{dI_{p}}{dt}=\left(1-q\right)\omega_{p}E_{p}-\gamma I_{p}\\ \frac{dA_{p}}{dt}=q\omega_{p}-\gamma^{\prime }A_{p}\\ \frac{dR_{p}}{dt}=\gamma^{\prime }A_{p}+\gamma I_{p}\\ \frac{dS_{m}}{dt}=ac\left(N-nI_{m}\right)-\left[\beta_{i}I_{i}+\beta_{pm}\left(A_{p}+I_{p}\right)\right]S_{m}-b_{m}\\ \frac{dE_{m}}{dt}=\left[\beta_{i}I_{i}+\beta_{pm}\left(A_{p}+I_{p}\right)\right]S_{m}-\left(\omega_{m}+b\right)E_{m}\\ \frac{dI_{m}}{dt}=\omega_{m}E_{m}+acnI_{m}-bI_{m}\\ \frac{dI_{i}}{dt}=-\varepsilon I_{i}-\beta_{i}S_{m}I_{i}\\ N_{m}=S_{m}+E_{m}+I_{m}\\ N_{p}=S_{p}+E_{p}+I_{p} \end{array}$$

where the seasonality parameter can be modeled by a trigonometric function, according to existing studies (12). We assume the following function for the parameter c:

$$\begin{array}{l} c=cos\;\left[\frac{2\pi \left(t-\tau \right)}{T}\right] \end{array}$$

### Parameter estimation

There are fifteen parameters in this model (β_*i*_, β_*pm*_, β_*mp*_, ω_*m*_, ω_*p*_, ε, γ, γ', *a, b, c, q, n*) according to the literature, see Table 2. The incubation period of dengue virus in human body is usually 4–8 days (9), therefore we selected the value of 6 days as the average, with ω_*p*_ = 0.1667. It usually takes 8–12 days for imported dengue virus to enter mosquito host and cause its onset (19), thus the value of 10 days was chosen in the model, with ω_*m*_ = 0.1000 per day. The proportion of asymptomatic infections is usually 68.75% (21), so *q* = 0.6875. The infectious period is 3–14 days (19, 20), we selected 7 days in the simulation, thus γ = γ' = 0.1429. Based on a previous study, the initial values of birth and mortality rate of mosquitoes was set as *a* = *b* = 0.0714 (19). In addition, with the constant change of resistance to mosquito-borne insecticide, we set the value range of *a* and *b* to 1/50–1/14 according to our study. The vertical transmission rate of dengue virus DENV-1 through vertical infection ranged from 1.4 to 17.4% (22), which was assumed it to be 10.0%, so *n* = 0.1000.

**Table 2:** Parameter definitions and values.

**Parameter**	**Description**	**Unit**	**Value**	**Range**	**Method**
*β_*i*_*	Transmission relative rate from input case to mosquitoes	1	5.11892 × 10^−6^	≥0	Curve fitting
*β_*pm*_*	Transmission relative rate from human to mosquitoes	1	8.01142 × 10^−6^	≥0	Curve fitting
*β_*mp*_*	Transmission relative rate from mosquitoes to human	1	1.24107 × 10^−5^	≥0	Curve fitting
*ω_*m*_*	Incubation relative rate of mosquitoes infection	Day^−1^	1/10	0.0833–0.1250	(19)
*ω_*p*_*	Incubation relative rate of human infection	Day^−1^	1/6	0.1250–0.2500	(9)
ε	Input case recovery ratio	1	0.1429	0.0714–0.3333	Curve fitting
γ	Removed relative rate of infectious individuals	Day^−1^	1/7	0.0714–0.3333	(19, 20)
γ′	Removed relative rate of asymptomatic individuals	Day^−1^	1/7	0.0714–0.3333	(19, 20)
*a*	Daily birth rate of mosquitoes	Day^−1^	1/14	0–1/14	(19, 20)
*b*	Daily mortality rate of mosquitoes	Day^−1^	1/14	0–1/14	(19, 20)
*c*	Seasonality parameter of the mosquitoes population	1	See text	0–1	Curve fitting
τ	Simulation delay of the initial time in the whole season	Day	242	≥0	Analysis on the reported data
*T*	Duration of the cycle	Day	365	≥0	Analysis on the reported data
*q*	Proportion of human asymptomatic infection	1	0.6875	0-1	(21)
*n*	Proportion of transovarial transmission	1	0.1	0.0140–0.1740	(22)

### Evaluate the risk of DF transmission in different conditions

We assessed the transmission risk of DF in Xiamen City through SEIAR model. According to practical experience and previous studies, we included the following factors as transmission risk factors into our research: community population, the number of imported cases, larvae density, adult vector density, mosquito insecticide resistance (23, 24). Consequently, in this study, BI method was used to assess the larvae density of the vector and light-trap method was used to measure assess the adult vector density. The changes in mosquito mortality reflect the intensity of mosquito-borne insecticide resistance.

According to the community situation of indigenous cases in Xiamen City in 2019, the community population was set as 10,000, 15,000, 20,000, and 25,000, and the parameter of imported cases were 1–60. Our previous studies on insecticide resistance have determined that the mosquito birth and mortality rate would be set as 1/50, 1/38, 1/26, and 1/14 (12, 23). Simultaneously, the number of susceptible mosquito-borne could be reflected by changing the mosquito density, which was 1, 2, 3, 4, and 5, respectively, in BI. Therefore, we have set the following scenarios to quantitatively assess the transmission risk of DF in Xiamen City:

Scenario 1: We set the community population as 10,000, and BI values were 1, 2, 3, 4, and 5. The mortality rates of mosquitoes were set as 1/50, 1/38, 1/26, and 1/14, respectively. And to see how many dengue cases were imported, which could cause the spread of indigenous secondary cases.
Scenario 2: We set the community population as 15,000, BI values were 1, 2, 3, 4, and 5, respectively; The mortality rates of mosquitoes were set as 1/50, 1/38, 1/26, and 1/14, respectively. And to see how many dengue cases were imported, which could cause the spread of indigenous secondary cases.
Scenario 3: We set the community population as 20,000, BI values were 1, 2, 3, 4, and 5, respectively; The mortality rates of mosquitoes were set as 1/50, 1/38, 1/26, and 1/14, respectively. And to see how many dengue cases were imported, which could cause the spread of indigenous secondary cases.
Scenario 4: We set the community population as 25,000, BI values were 1, 2, 3, 4, and 5, respectively; The mortality rates of mosquitoes were set as 1/50, 1/38, 1/26, and 1/14, respectively. And to see how many dengue cases were imported, which could cause the spread of indigenous secondary cases.

### Simulation and statistical analysis

We used Berkeley Madonna ver.8.3.18 (developed by Robert Macey and George Oster of the University of California at Berkeley, CA, USA) for parameter fitting and model simulation. The goodness-of-fitting was assessed by least root-mean-square error between simulated and observed number of new indigenous cases per day between August 24th and November 5th. The simulation method was the Runge–Kutta method of order four. Differential equations were solved by the step of 0.02. Meanwhile, the goodness of fit was judged by the coefficient of determination (*R*^2^) value.

## Results

### Epidemiological characteristics of DF in Xiamen City

**Figure 2:**
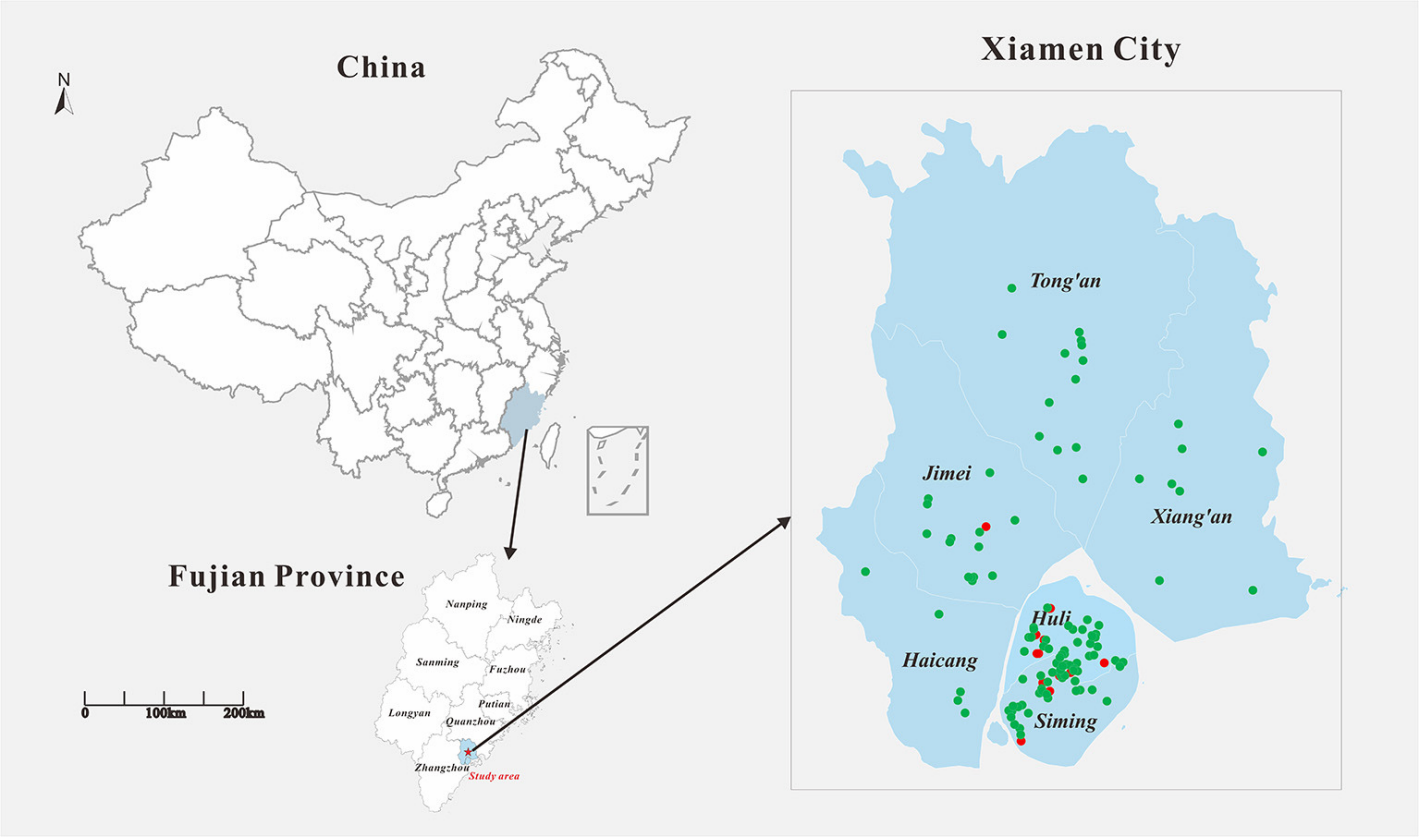
Geographical location of the study area and distribution of DF cases in Xiamen in 2019. Filled red circle represents the indigenous cases and filled green circle represents the imported cases.

**Figure 3:**
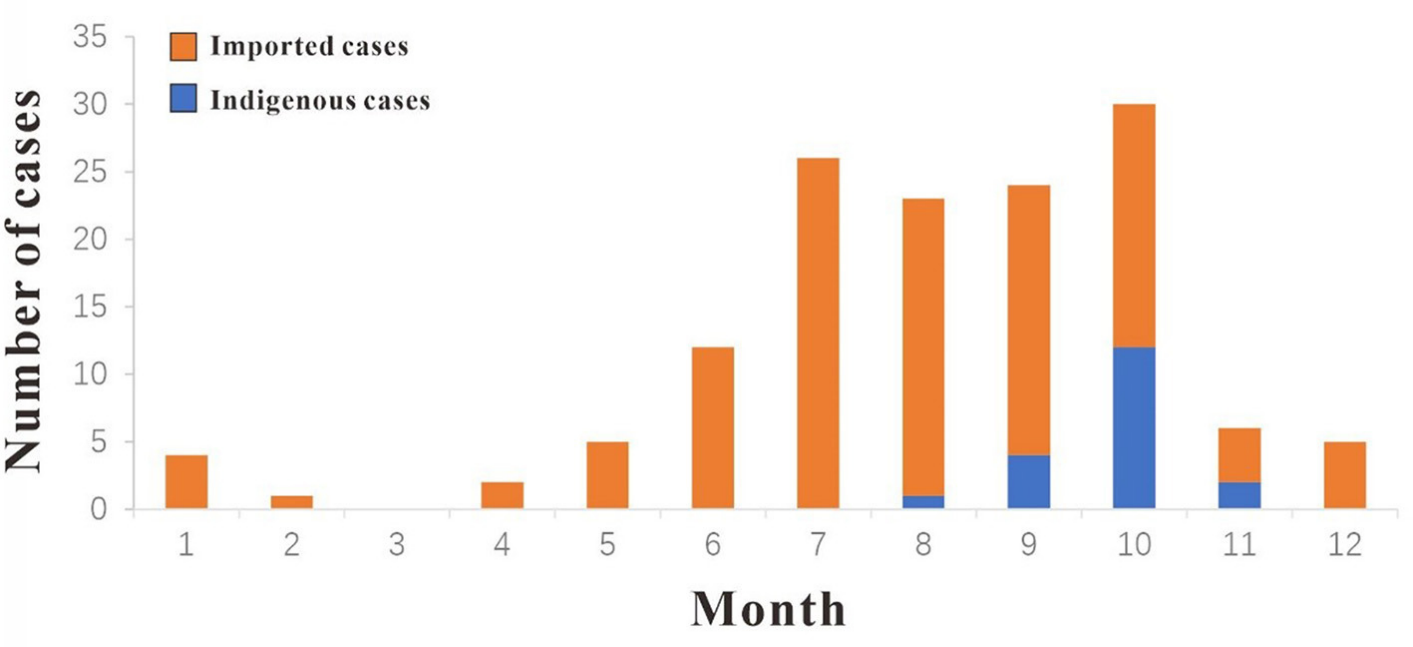
Reported cases of DF in Xiamen City, P.R. China, in 2019.

In 2019, a total of 138 cases of DF were reported in Xiamen City, and there were 19 indigenous cases and 119 imported cases. The distribution of all DF cases in Xiamen City is shown in Figure 2, among which indigenous cases were mainly reported in Huli District, Siming District and Jimei District. But the imported cases were reported in all districts of Xiamen City. The number of new cases in Xiamen City per month is shown in Figure 3. The first indigenous case was reported in the Jinshan community of Huli District on August 24th, 2019. The peak of DF incidence was from July to October, and the number of DF cases reported in these 4 months accounts for 75% of the total reported cases in the whole year. The population distribution of DF cases in Xiamen City in 2019 is shown in Table 3. Male patients predominate in imported cases, while females outnumber males in local cases. Meanwhile, most of the reported cases of DF were most reported in people aged between 20 and 50 years old, with no cases reported in people younger than 10 years old. In addition, a higher proportion of cases were reported in commercial services and domestic activities.

**Table 3:** The population distribution of DF cases in Xiamen City in 2019.

		**Indigenous cases**	**Imported cases**
Gender	Male	8	94
	Female	11	25
Age	≤10	–	–
	11–20	2	4
	21–30	6	39
	31–40	2	39
	41–50	5	34
	51–60	1	2
	≥61	3	1
Occupation	Commercial service	7	21
	Student	2	4
	Staff member	3	1
	Worker	2	2
	Housework and unemployment	4	18
	Farmer	–	1
	Catering food industry	–	7
	Others	1	64

### Investigation on mosquito-borne ecology and insecticide resistance monitoring

**Figure 4:**
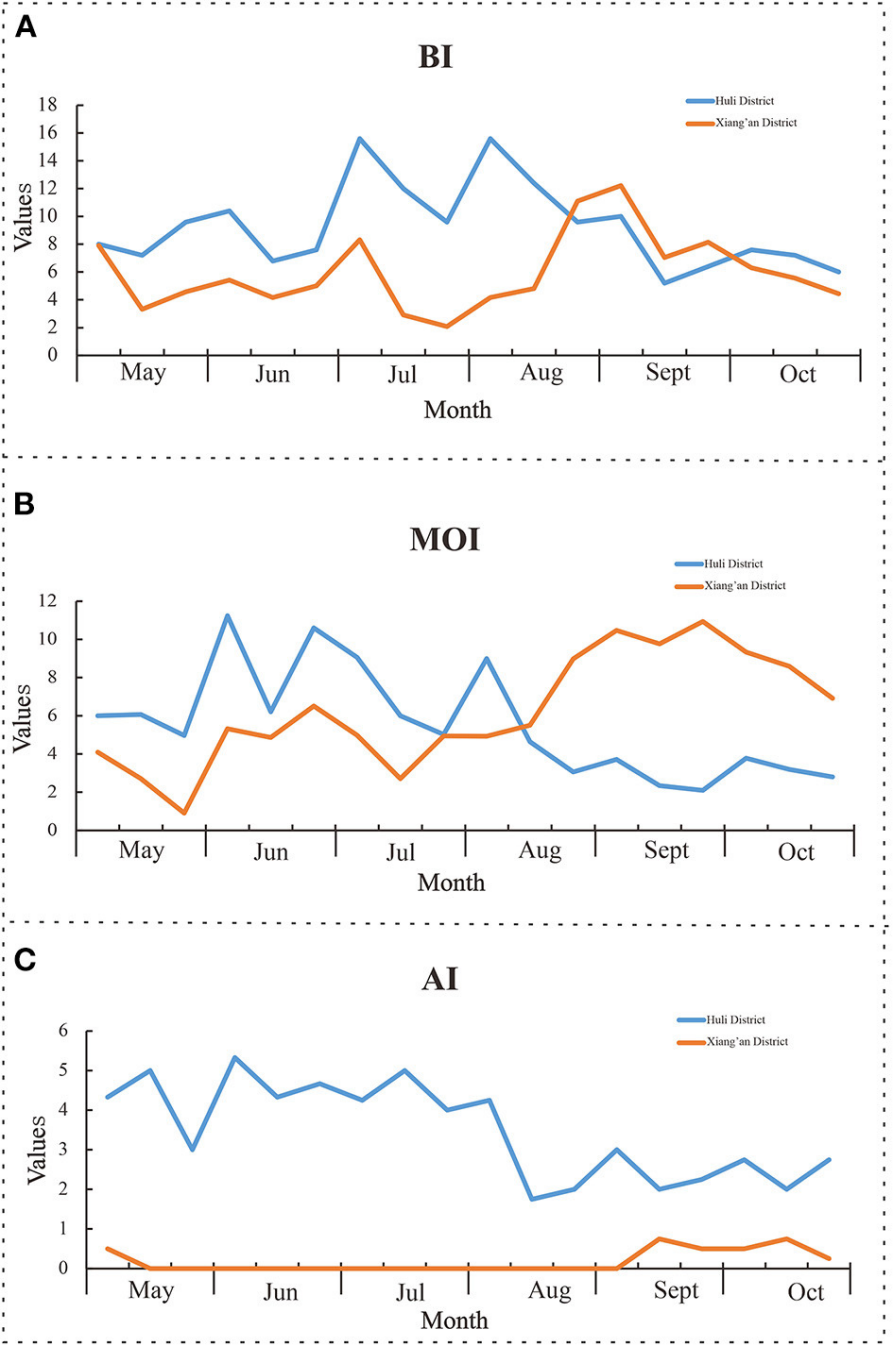
Monitoring results of mosquito-borne ecology. **(A)** Brayton index; **(B)** container index; **(C)** human-baited double net trapping.

We conducted a household survey of *Ae. Albopictus* between May and October 2020. A total of 9,060 *Ae. Albopictus* were captured, and 693 Aedes larvae were positive in water accumulation of Aedes larvae were found, see Figure 4A. The average value of BI is 7.6, among which the average value of BI in Huli District is 9.3 and that in Xiang'an District is 6.1. The monitoring results of BI are shown in Table 3. It is noticeable that the BI index of Xiamen City is higher than the threshold value of 5 from May to October 2020. Besides, from May to October 2020, we have placed a total of 9,840 mosquito container in Xiamen City. Among them, 8,893 were recovered with rate of 90.4%. The results showed that there were 518 positive traps, and the average index of CI was 5.8, which was higher than the safety level of 5 from June to October. Therefore, it can be found that the change trend of CI is similar to that of BI, its the value is generally lower than BI, see Figure 4B.

For the monitoring of adult mosquito, we have the Human-baited double net trapping technique. From May to October, a total of 276 double-layered accounts were deployed. Finally, it was calculated that 1.7 mosquito nets were detected per hour, of which the highest was 2.2 in July, and the threshold value was higher than 2 in both June and July, see Figure 4C.

Therefore, we monitored the insecticide resistance of *Ae. albopictus* in Xiamen City, the results demonstrated that knock-down rate of mosquitoes at 1 h, the corrected mortality rate for 24 h and the insecticide resistance are shown in Table 4. Furthermore, the resistance times and resistance result of *Ae. albopictus* larvae exposed to parathion, prepoxur and pyriproxyfen are shown in Table 5. The adult *Ae. albopictus* in Huli District is sensitive to insecticides such as oxymoron, fenitrothion, residual carbofuran, malathion and chlorpyrifos, and high resistance to pyrethroid insecticides such as deltamethrin, high efficiency cypermethrin and high efficiency cyfluthrin. The larvae of *Ae. albopictus* are sensitive to disulfiram, with low resistance to residual carbofuran and medium resistance to pyriproxyfen. Adult *Ae. albopictus* is sensitive to oxytetracycline, fenitrothion and chlorpyrifos, possibly resistant to residual chlorpyrifos and malathion, and highly resistant to deltamethrin, cypermethrin, deltamethrin, cypermethrin and other pyrethroid insecticides in the Xiang'an District. Larvae of *Ae. albopictus* are sensitive to disulfoton and residual chlorpyrifos and moderately resistant to imidacloprid.

**Table 4:** Insecticide resistance of *Ae. albopictus*.

**Pesticides**	**(Adjusted) mortality rate**		**Population determine**	
	**Huli**	**Xiang'an**	**Huli**	**Xiang'an**
0.2% bendiocarb	100%	100%	Sensitive species	Sensitive species
0.2% fenitrothion	96%	99.24%	Likely resistant species	Sensitive species
0.03% deltamethrin	93%	24.07%	Likely resistant species	Resistant species
0.04% permethrin	64%	53.57%	Resistant species	Resistant species
0.5% propoxur	100%	95.71%	Sensitive species	Likely resistant species
0.5% malathion	99%	99.31%	Sensitive species	Sensitive species
0.08% beta-cypermethrin	38%	30.89%	Resistant species	Resistant species
0.07% lambda-cyhalothrin	75%	66.67%	Resistant species	Resistant species
2% chlorpyrifos	100%	100%	Sensitive species	Sensitive species
0.4% beta-cypermethrin	43%	68.87%	Resistant species	Resistant species

**Table 5:** Insecticide resistance of *Ae. albopictus* larvae.

**Pesticide**	**Population**	**Regression equation**	**LC50 (mg/L)**	**95% CI**	**Resistance multiple**
Disulphion	Sensitive strain	*y* = 21.47 + 8.71*x*	3.43 × 10^−3^	3.31 × 10^−3^, 3.55 × 10^−3^	
	Huli	*y* = 9.65 + 4.1*x*	4.42 × 10^−3^	4.12 × 10^−3^, 4.71 × 10^−3^	1.29
	Xiang'an	*y* = 9.17 + 4.13*x*	5.98 × 10^−3^	5.57 × 10^−3^, 6.48 × 10^−3^	1.74
Propoxur	Sensitive strain	*y* = 7.23*x* – 0.15	1.05	0.95, 1.13	
	Huli	*y* = 4.62*x* – 2.38	3.27	3.08, 3.49	3.12
	Xiang'an	*y* = 3.33*x* −1.64	3.11	2.84, 3.42	2.97
Pyripropoxyfen	Sensitive strain		1.01 × 10^−5^	6.24 × 10^−6^, 1.45 × 10^−5^	
	Huli	*y* = 1.907 + 0.531*x*	2.57 × 10 × 10^−4^	1.54 × 10^−4^, 4.03 × 10^−4^	25.55
	Xiang'an	*y* = 2.837 + 0.740*x*	1.48 × 10^−4^	9.4 × 10^−5^, 2.24 × 10^−4^	14.71

### Risk assessment of different transmission models

**Figure 5:**
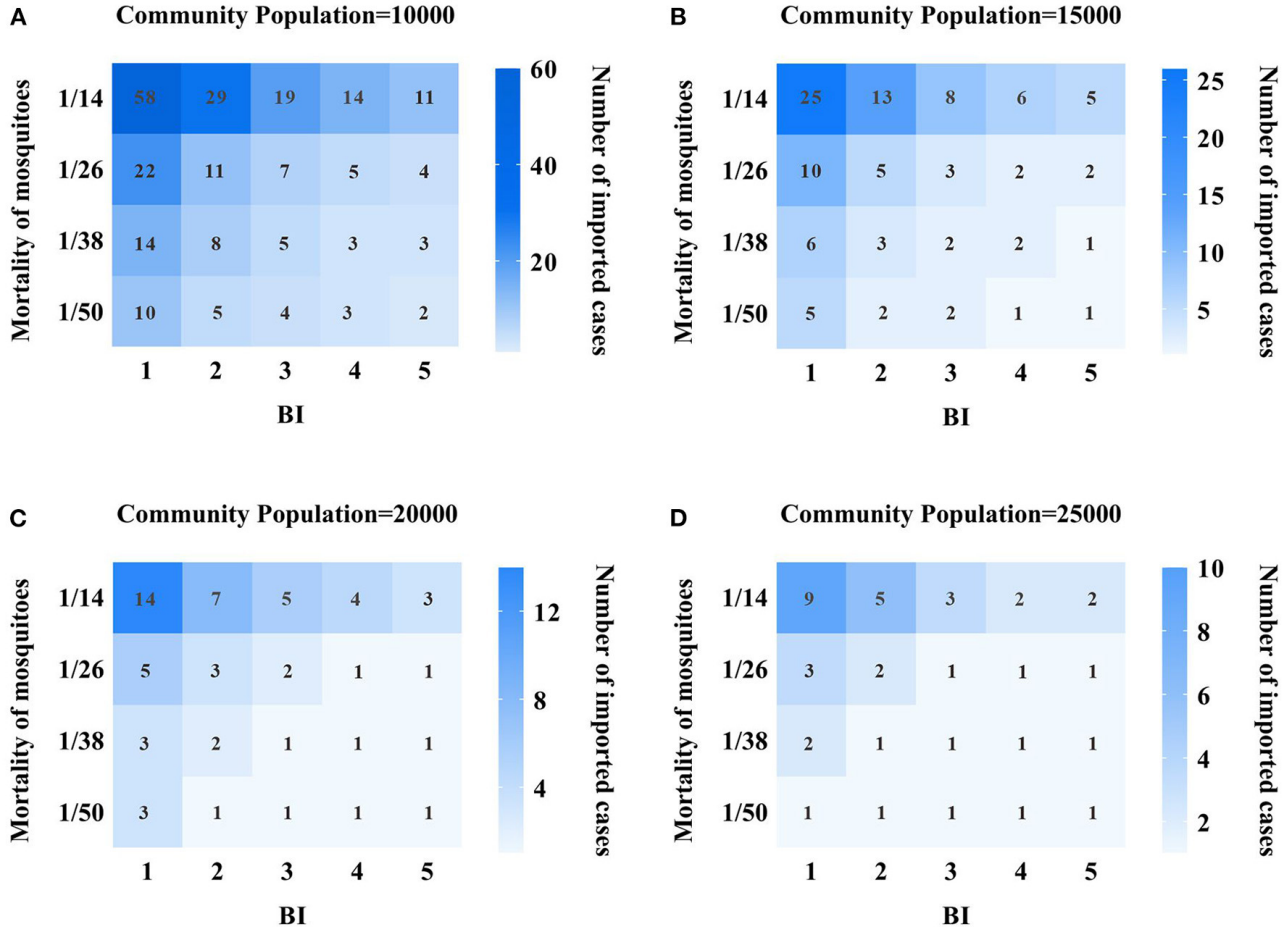
Quantitative assessment results of DF transmission risk in Xiamen City. **(A)** 10,000 population community; **(B)** 15,000 population community; **(C)** 20,000 population community; **(D)** 25,000 population community; the vertical index represents the mortality of mosquitoes, and the value is 1/15, 1/26, 1/38, and 1/50 respectively, and the horizontal indicator represents the value of BI, which are 1, 2, 3, 4, 5, respectively. The values in each grid represent the number of dengue cases that need to be imported to cause local transmission, and the darker the grid, the more dengue cases need to be imported.

In this study, by adjusting different parameters in the model, we observed whether different imported cases can cause the transmission of indigenous DF cases, and quantitatively assessed the transmission risk of DF in Xiamen City, as shown in Figure 5. In scenario 1, we set the community population to 10,000. At this point, by changing BI value, birth rate and mortality rate of mosquitoes, we found that 58 DF cases imported from the community can cause indigenous DF cases when mosquito mortality rate is 1/14 and BI value is 1. With the BI values changing from 2, 3, 4–5, the imported case parameters are set to 29, 19, 14, and 11 cases, respectively, which can generate indigenous secondary cases. On the other hand, when the BI value is fixed at 1, the mosquito mortality rate changes to 1/50, so it takes 10 imported cases to cause the spread of indigenous cases. However, when changing the birth rate parameters, the impact on the model is limited, and the input of dengue cases within the set range cannot produce local secondary cases. As mosquito mortality declines and BI value increases, so did the number of imported cases that can cause the spread of local dengue cases, see Figure 5A.

In scenario 2, the community population is 15,000. At this time, the BI value is set to 1, the mosquito mortality rate is 1/14, and 25 imported cases are needed to produce local secondary cases. However, when the mortality rate is 1/14 and the BI value is 5, or when the mortality rate is 1/50 and the BI value is 1, only five imported cases are needed to produce local secondary cases. At the same time, we also found that when the BI value increased, the mortality dropped to a certain range, and only one imported case could cause local dengue fever cases, and the risk of DF transmission was relatively high at this time, see Figure 5B.

In Scenarios 3 and 4, the community population reaches 20,000 and 25,000, respectively. The results showed that under the same BI value and mosquito mortality conditions, the larger the community population, the smaller the number of imported cases needed to cause local dengue fever transmission, and the higher the transmission risk. Moreover, we changed the BI value and mosquito mortality, and found that the lower the mortality rate, the higher the BI, the higher the transmission risk of dengue fever. Especially when the BI value is above 3 and the mortality rate is < 1/26, only one dengue fever case is needed to produce an indigenous DF case, see Figures 5C, D.

## Discussion

DF cases was first reported in Foshan city in China in 1978. The dengue virus then spread widely to Guangdong, Guangxi, Hainan and Fujian province, causing hundreds of thousands of patients (25). As a main economic city in Fujian Province, Xiamen has a suitable climate and a large population mobility, resulting in a heavy public health burden caused by DF. In this study, we used the transmission dynamics model to quantitatively evaluate the influence of community population, mosquito density and mosquito-borne insecticide resistance on DF transmission in Xiamen City, so as to promote the formulation of more effective prevention and control strategies.

### Population risk assessment

In 2019, the distribution of DF cases in Chinese mainland has expanded significantly, with 1,066 regions reporting imported cases and 550 regions reporting indigenous cases (26). From 2005 to 2019, the imported cases of DF in Xiamen City showed a rapid upward trend. In 2019, Xiamen reported a total of 119 imported cases from multiple regions and countries, mainly distributed in Huli and Siming District. The public health and epidemic prevention departments of these two administrative districts need to pay extra attention to the transmission of imported cases to secondary cases in the local areas. The indigenous cases were reported mainly from August to October, for the main reason may be that the temperature and humidity in summer are suitable for mosquito-borne growth and reproduction, leading to the local spread of dengue virus (12, 27). Imported cases have also been reported in 11 months except March. We believe that Xiamen City, as a coastal city, has frequent communication with Southeast Asian countries and regions, and there are many imported cases reported during the high incidence of DF in summer. Accordingly, the customs and epidemic prevention departments should pay attention to the movement of personnel from areas with high incidence of DF and do a good job of port education on DF prevention, which can effectively reduce the local spread and even outbreak of DF. The reported cases are mainly concentrated in the middle-aged and young people aged 21–50, and the majority of them are professional engaged in commercial services. According to the study, these people are more susceptible to DF infection due to their active work. Therefore, the education and medical investigation of these people should be strengthened as part of daily prevention work.

### Mosquito vector risk assessment

According to our research, we selected Huli District and Xiang'an District of Xiamen City to investigate mosquito-borne ecology and monitor insecticide resistance. The results showed that the average value of BI and CI in Huli District were 9.3, 5.3, then 6.1 and 6.4 in Xiang'an District, all of which were higher than the safety level of 5. In this cases, larvae proliferated rapidly, increasing the risk of dengue virus transmission (27). At this point, when imported cases occur in the jurisdiction, DF might spread or even spread locally. At the same time, we measured the number of adult mosquitoes by HDN method, and the results showed that the average of HDN in Huli District was 3.4, which gradually decreased after August, while the HDN in Xiang'an District was always at a low level, just similar to the number of indigenous cases. As previously monitored, *Ae. albopictus* in Xiamen is sensitive to malathion and propoxur, and has high resistance to pyrethroid insecticides such as deltamethrin, beta-cypermethrin, and beta-cypermethrin. Thus, we selected mosquitoes in two districts to carry out insecticide resistance tests. The results illustrated that *Ae. albopictus* in Huli District was sensitive to warfarin, fenitrothion, propoxur, malathion, and chlorpyrifos, and had higher resistance to other insecticides. *Ae. albopictus* in Xiang'an District was only sensitive to warfarin, fenitrothion, and chlorpyrifos, showing different resistance levels to other insecticides. Based on previous studies, mosquito resistance to chemical insecticides is inevitable, so we need to avoid using chemical insecticides blindly (28, 29). The local public health authorities can scientifically assess the risk of mosquito-borne transmission on the basis of experiments, rationally select sensitive and low-resistance pesticides, and use them in crop rotation on an annual basis. This can effectively present the increase of resistance and achieve the purpose of reducing mosquito density.

### Model evaluation

In this study, the ODE dynamics model was used to investigate the impact of potential risk factors such as community population, number of imported cases, mosquito vector density and mosquito insecticide resistance on the local transmission of DF in Xiamen. We set up four scenarios with simulated community population of 10,000, 15,000, 20,000, and 25,000, and input different numbers of DF cases to quantitatively evaluate the spread of indigenous DF cases. In the model, the density of mosquito vectors is reflected by the change of BI index, while the insecticide resistance of mosquitoes is assumed to change the birth rate and mortality rate of mosquitoes. According to our simulation results, when the community population is 10,000, the mortality rate of mosquitoes is 1/14 of the highest value in the set range, and the BI index is 1, both insecticide resistance and mosquito vector density of mosquitoes are at the lowest value, so 58 imported DF cases are needed to cause the spread of indigenous DF cases. As the density of mosquito vectors gradually increases, the number of imported cases required for indigenous DF transmission gradually decreases. Similarly, when the mortality rate of mosquitoes is reduced to 1/50, that is, mosquito resistance to insecticide is very high, the results show that only 10 imported cases can result in secondary local cases. This result suggests that public health departments should do a good job in killing mosquito vectors and choose sensitive and low-resistance chemical. In addition, in the model, we found that the birth rate of mosquitoes has little influence on the model, when we change the birth rate parameters, a certain number of dengue cases are input within the set range, which cannot cause the spread of indigenous cases.

When the community population gradually increased to 15,000, 20,000, and 25,000, the results of model simulation showed that when the mosquito mortality rate was 1/14 and BI value was 1, the number of imported cases needed to cause indigenous DF cases was 25, 14, and 9, respectively, that is, when mosquito-borne insecticide resistance and mosquito-borne density were consistent, the community population base had a great influence on local transmission of DF. Therefore, CDC should do well in health education and health prevention and control in large communities to effectively improve the prevention and control effect of DF. In the meantime, we found that when the community population is more than 20,000, the mosquito density is more than 3, and the mortality rate of mosquitoes is < 1/38, only one case can be imported, which can cause the spread of indigenous DF. At this time, the risk of transmission is very high, so mosquito prevention and control work mainly in large communities is needed to prevent the synergistic effect of these key factors from causing the spread of indigenous DF.

The prevention and control of dengue fever is a social work, that requires the participation of health and epidemic prevention departments and the public. For places where DF is less threatening, we can mobilize the masses to carry out environmental prevention and control measures through community departments, so as to reduce the mosquito density in the external environment. For places where DF is a great threat, we suggest that public health departments should adopt chemical control during the high incidence period of DF (July to October) to reduce the risk of DF transmission, and select sensitive and low-resistance chemical insecticides to reduce the risk of DF transmission.

## Limitation

It is worth noting that there are some limitations to our research. First of all, infectious disease outbreak assessment includes three steps and two factors, but in this study, we did not discuss the impact of environmental factors on mosquito vectors. Secondly, because DF is transmitted through vector, we can't evaluate the transmission ability of dengue fever by calculating *R*_0_ (the basic reproduction number) or *R*_*eff*_ (the effective reproduction number). In the future work, we will also explore a feasible way to solve these problems.

## Conclusions

In this study, propagation dynamics model was used to assess and predict the risk of DF. We emphasize that imported cases, community population, mosquito density and insecticide resistance play a key role in local DF transmission. Mosquito insecticide resistance has been identified as the most critical factor for evaluating DF communication risks and implementing management control measures. So public health authorities in China should pay more attention to mosquito control. The change of mosquito-borne birth rate and mortality rate can be regarded as indicators of mosquito-borne insecticide resistance. We suggest that detection of threshold effects of the number of imported cases, mosquito density and the changes of birth rate and mortality rate of local mosquito vectors tested by the transmission dynamics model can be used to predict and evaluate the risk of dengue fever epidemic. The identified factors are beneficial to the establishment of early warning and monitoring system of infectious diseases.

## Data availability statement

The original contributions presented in the study are included in the article/Supplementary material, further inquiries can be directed to the corresponding authors.

## Author contributions

TC, ZG, WL, and XL designed research. ZG, WL, XL, BA, SiW, BD, TY, JH, LLe, and ZZ analyzed data. WL, ZG, BA, LLu, and SiW completed the experiment. TC, ZG, WL, XL, BA, BD, LLe, ZZ, ZL, PL, CL, and LLu conducted the research and analyzed the results. TC, ZG, WL, XL, BA, and LLu wrote the manuscript. All authors read and approved the final manuscript.
