# Supplementary material for: Model-based risk assessment of dengue fever transmission in Xiamen City, China
Source: Front Public Health. 2023 Feb 13;11:1079877. doi: 10.3389/fpubh.2023.1079877 (PMC9969104; doi:10.3389/fpubh.2023.1079877)
Supplement: Supplementary file 1 [file Data_Sheet_1.docx]

**Text S1. Supplementary methods.**

I. Larval mosquito monitoring plan

1. Brayton index (BI) method

Huli district and Xiang 'an District of Xiamen City were selected as monitoring sites. According to different geographical orientation of each administrative region, four streets in urban residential areas were selected for monitoring, with a total of no less than 100 households; four townships (towns) in rural residential areas shall be selected for monitoring, with a total of no less than 100 households. Other habitats in urban areas such as parks and bamboo forests, used tire dump sites, scrap stations, construction sites etc. would be monitored by BI method at the same time, and the area will be converted according to the definition of households as follows, with a total investigation of no less than 50 households.

During the investigation, check and record the breeding situation of Aedes mosquito larvae in all indoor and outdoor water containers or water bodies. The larvae were collected for species identification or reared in the laboratory until they reached adulthood for species identification. BI was calculated and the monitoring results were filled in the record table (see Table 1). Monitoring devices include flashlights, spoons, straws, mosquito collection devices and labels.

The definition of household is consistent with the *Ae.albopictus* Surveillance Guidelines (2014), that is, for each family, dormitory, office, hotel, 2 rooms for a household. And for farmers' market, greenhouse, external environment, indoor public places and so on every 30*m^2^* is defined as one household.

The monitoring time was set from May to November, and the monitoring work was carried out three times a month at each monitoring site, with rain and rain postponed. The following density index formula is used to calculate Brayton index (BI) :

$$BI=\frac{Number of positive containers of Ae.albopictus}{Number of households surveyed}\times100\%$$

2. Container index (CI) method

Huli and Xiang 'an District of Xiamen City were selected as monitoring sites. The districts and townships where the CI method was carried out were the same as those where the BI method was carried out, and the CI method was carried out first.

According to different geographical orientation, at least 100 mosquito CI were placed in 4 streets in urban residential areas, and at least 100 mosquito CI were placed in 4 townships (towns) in rural residential areas, totaling 200 mosquito containers. In addition, at least 50 containers will be placed in parks and bamboo forests and used tire storage sites, waste stations, construction sites in the urban areas of the monitoring sites.

In the investigation, an container was placed at a distance of 25-30 meters for 4 consecutive days, and the adult mosquitoes were collected for inspection on the fourth day. Mosquito eggs were raised to old larvae or adult mosquitoes for species identification, mosquito container index was calculated, and the monitoring results were filled in the record form and summary table (See Table 2 and Table 3). Monitoring devices include mosquito container, white filter paper, overnight tap water, label paper, etc.

The monitoring time was set from May to November, and the monitoring work was carried out three times a month at each monitoring site, with rain and rain postponed. The following density index formula was used to calculate the mosquito container index (CI) :

$$CI=\frac{Number of positive containers}{Number of effective containers}\times100\%$$

II. Adult mosquito surveillance plan

Adult mosquitoes were monitored in Huli district and Xiang 'an District of Xiamen city by human-baited double net trapping method. In each administrative region, one site was selected for each of the four habitats: urban residential area, rural residential area, park/bamboo forest, used tire dump site/scrap station/construction site, and two tents were made for each site, with an interval of more than 100 meters.

Mosquito nets were placed in sheltered and shady places in each habitat, and people were kept away from the work. During the peak period of Aedes mosquito activity in the afternoon (15:00-18:00), the attractors exposed their lower legs in the internal closed mosquito net, and the collectors wore long clothes and pants. Mosquito repellents were not used in the monitoring process. To use electric mosquito device quickly gather perched between two layers of bed nets in *Ae.albopictus* net on the media, and then leave as soon as possible, then monitor lasted 30 minutes. All the trapped *Ae.albopictus* should be collected. The mosquito vectors collected by each mosquito net should be kept separately and brought back to the laboratory for freezing and death. The species, gender and counting should be identified and filled in the record form (see Table 4). After identification, the specimens should be stored in the refrigerator below -70℃. Monitoring equipment adopts double laminated tent, counter, flashlight and electric mosquito suction device.

The monitoring time was set from May to November, and the monitoring work was carried out three times a month at each monitoring site, with rain and rain postponed. The following density index formula is used to calculate the human-baited double net trapping method(HDN).

$$HDN=\frac{Number of female mosquitoes captured}{Number of mosquito nets\times30\mathrm{minutes}}\times60minutes$$

III. Mosquito-borne resistance surveillance plan

1. Collection and feeding of mosquitoes
2. Selection of collection sites

Huli district and Xiang 'an District of Xiamen City were selected as monitoring sites to collect mosquitoes.

1. Mosquito collection and feeding

The worms were collected from different directions of east, south, west, north and middle of each monitoring site. According to the items listed in Table 5, the collection time, location, latitude and longitude (or geographic coordinates), the number and state of the collected worms were recorded. After the samples were collected, the species were identified according to the breeding characteristics of mosquitoes and morphological characteristics of adult mosquitoes or the 4th instar larvae. The same monitoring site was mixed and raised in different directions, and different monitoring sites were raised separately. F1 generation was used to measure drug resistance.

1. Surveillance of larval resistance
2. Monitoring methods: The impregnation method recommended by WHO (refer to GB/T26347-2010) was used to measure the LC50 value of larvae and calculate the resistance ratio (RR).
3. Test requirements: late 3rd instar to early 4th instar larvae
4. Insecticide for testing

| **Insecticide** | **Pesticide** |
| --- | --- |
| Organophosphate | Disulphion |
| Propoxur | Propoxur |
| Biological | Pyriproxyfen |
|  | Bt.i |
|  | S - olefin ester |

*The insecticide raw materials were uniformly provided by the National Institute of Infectious Diseases, China CDC

1. The determination of steps
2. 5-7 series of insecticide concentrations prepared with acetone;
3. Take a corresponding number of beakers, add 200mL dechlorination water to each, and siphon off 100µL with a micropipette;
4. First add 100µL acetone into the beaker of the control group, and then add 100µL of various concentrations of liquid medicine to the experimental group in turn, and stir evenly with a glass rod or magnetic stirrer in accordance with the sequence of control, low concentration and high concentration. Set at least 3 repetitions for each concentration;
5. Use a larval pipette to absorb the late 3rd to early 4th instar larvae, and filter out the water with a small colander. Add 20-30 larvae into each beaker according to the sequence from control group, low concentration group to high concentration group;
6. The mosquitoes were placed in an incubator or room with a set temperature of (25±1) ℃ and humidity of (70±10) % for 24 hours (pyripropyl ether was observed until the emergence or death of the control group) to check the death situation of mosquitoes;
7. Results and drug resistance level

The determination information and results were recorded in Table 6. Virulence regression line, median lethal concentration (LC50) (IE50 of eclosion inhibition was calculated by pyripropyl ether), slope value, Chi-square value and other data were obtained to calculate resistance ratio (RR).

RR= LC50 value of the tested population/LC50 value of the sensitive population (or IE50 value of the tested population/IE50 value of the sensitive population). The level of resistance can be determined by referring to the following criteria: RR < 3 is sensitive, 3≤RR < 10 is low resistance, 10≤RR < 40 is moderate resistance, and RR≥40 is high resistance. (LC50/IE50 values of sensitive strains were uniformly provided by Institute of Infectious Diseases, China CDC)

1. Surveillance of drug resistance in adult mosquitoes
2. Monitoring methods: The mortality rate of adult mosquitoes at diagnostic dose was determined by the contact tube method recommended by WHO (GB/T26347-2010);
3. Test requirements: 3~5 days after emergence of female mosquitoes without blood sucking;
4. Insecticide for testing

| **Insecticide** | **Pesticide** |
| --- | --- |
| Pyrethroids | Deltamethrin |
|  | Permethrin |
|  | Beta-cypermethrin |
|  | lambda-cyhalothrin |
| Carbamate | Propoxur |
|  | Bendiocarb |
| Organophosphate | Malathion |
|  | Fenitrothion |
|  | Chlorpyrifos |

*The insecticide raw materials were uniformly provided by the National Institute of Infectious Diseases, China CDC

1. The determination of steps
2. The recovery tube was connected to the partition board, and 20-30 healthy female mosquitoes that did not suck blood for 3-5 days after emergence were artificially absorbed by mosquito tube and placed in the recovery tube in parallel for 15min to remove unhealthy mosquitoes;
3. Fitted on the other side of the diaphragm has contact line stick medicine paper tube (tube cleaning in order to contact convenient, can be put in the medicine paper before cushion layer and the medicine paper size consistent clean paper), the recovery cylinder in the following, upright, gently pat to restore mosquito gathered in the bottom of the barrel, then instantaneous clapboard out, upside down contact cylinder and recovery cylinder position, Gently blow the mosquito in the recovery cylinder into the contact cylinder and quickly close the separator;
4. Lay the tube flat, blow the mosquito back to the recovery tube after contact for 1 hour, remove the contact tube, and record the knockdown number of pyrethyl insecticides after contact for 1 hour;
5. Place cotton balls soaked in 10% sugar water above the recovery cylinder and check the results 24 hours later.
6. Criteria for determining the death of test insects

The test insect is considered dead if it does not move at all, or only its body, feet, wings or antennae vibrate without possibility of survival. The test results are recorded in Table 7.

1. Results and drug resistance level

Mortality = (number of dead worms/total number of tested worms) ×100%

When the mortality rate of the control group is less than 5%, no correction is required. The mortality rate of the control group is between 5% and 20%, and the Abbott formula is used for correction. If the mortality rate of the control group is more than 20%, the test will be regarded as invalid and re-measured.

Adjusted mortality =100%* (mortality in treatment group - mortality in control group) / (1- mortality in control group)

The criteria of resistance level were as follows: the mortality rate of mosquitoes at diagnostic dose was 98% ~ 100%, indicating that mosquitoes were sensitive species. When the mortality rate was 80% ~ 98% (excluding), it indicated that the population was possibly resistant. Mortality rates<80% indicates that it is a resistant population.

**Table1 Brayton index method monitoring**

| Time： Year Month Day  Place： Province (Autonomous Region/Municipality) City(county) Township (street) Village (Neighborhood Committee)  Environment type: Urban □; Rural □; Hospital □; Park □; Construction Site □; Salvage Station □; Tire factory □; Port/Wharf □; Others □ | | | | | | | | | | | | | | |
| --- | --- | --- | --- | --- | --- | --- | --- | --- | --- | --- | --- | --- | --- | --- |
| number | adress | Survey site (indoor/outdoor) | ^a^ Water | | | | | | | | | ^b^ the Larvael | | |
|  |  |  | Bonsai, aquatic plants | reservoir tub,  basin, barrel, altar, trough | Idle containers (bowls, bottles, jars, cans) | Open channel, rockery pool | Bamboo head, tree hole, stone hole | Tires, used tires | Green belt garbage, water-reservable waste | Basement and car park | Other water bodies | Aedes | Anopheles | culex |
|  |  |  |  |  |  |  |  |  |  |  |  |  |  |  |
|  |  |  |  |  |  |  |  |  |  |  |  |  |  |  |
|  |  |  |  |  |  |  |  |  |  |  |  |  |  |  |
|  |  |  |  |  |  |  |  |  |  |  |  |  |  |  |
|  |  |  |  |  |  |  |  |  |  |  |  |  |  |  |
|  |  |  |  |  |  |  |  |  |  |  |  |  |  |  |
| Brayton index (BI): | | | | | | | | | | | | | | |

Name of Organization: Monitor: Reviewer:

^*a^ water: fill in one record for each water body type; ^b^ the Larvae: type the corresponding section with “√”。

**Table 2. Container monitoring**

| Time： Year Month Day  Place： Province (Autonomous Region/Municipality) City(county) Township (street) Village (Neighborhood Committee)  Weather: Sun □ Cloudy □ Rain □ Temperature ℃, Top ℃，Lowest ℃ Relative Humidity: %  The geographical location of street or village: Longitude Latitude | | | | | |
| --- | --- | --- | --- | --- | --- |
| Place | Mosq-Ovitrap | | | | |
| Unit and Address | number | *Ae. Albopictus* eggs (+/-) | Aedes larvae (+/-) | *Ae. Albopictus* (+/-) | The total number of positive |
|  |  |  |  |  |  |
|  |  |  |  |  |  |
|  |  |  |  |  |  |
|  |  |  |  |  |  |
|  |  |  |  |  |  |
|  |  |  |  |  |  |
|  |  |  |  |  |  |
|  |  |  |  |  |  |
|  |  |  |  |  |  |

* "+" means that the ovitrap examined has found aedes mosquito eggs, larvae or adults

**Table 3 Container index monitoring**

| Number | Environment  (Inside/Outside) | Place | Number of mosquito containers | Number of effective mosquito container devices | Number of positive eggs of Aedes mosquitoes | Positive number of Aedes larvae | Positive number of adult Aedes mosquitoes | Total number of positive mosquito containers | Mosquito container index |
| --- | --- | --- | --- | --- | --- | --- | --- | --- | --- |
|  |  |  |  |  |  |  |  |  |  |
|  |  |  |  |  |  |  |  |  |  |
|  |  |  |  |  |  |  |  |  |  |
|  |  |  |  |  |  |  |  |  |  |
|  |  |  |  |  |  |  |  |  |  |
|  |  |  |  |  |  |  |  |  |  |
|  |  |  |  |  |  |  |  |  |  |
|  |  |  |  |  |  |  |  |  |  |
|  |  |  |  |  |  |  |  |  |  |
| Name of Organization: Monitor: Reviewer:  Date: | | | | | | | | | |

*Mosq-Container index = number of positive adult or eggs of Aedes/number of effective Mosq-Conatiner ×100

**Table 4 Double stack method monitoring record form**

Time: Year Month Day

Place: Province (Autonomous Region/Municipality) City(county) Township (street) Village (Neighborhood Committee)

Relative Humidity: ％ Wind Speed: m/s

Weather: Sun □ Cloudy □ Rain □

Temperature ℃, Top ℃, Lowest ℃

| Place | Environment* | Starting Time | Ending Time | Aedes albopictus | | Aedes aegypti | | Accounting Index  (per hour) |
| --- | --- | --- | --- | --- | --- | --- | --- | --- |
|  |  |  |  | Female | Male | Female | Male |  |
|  |  |  |  |  |  |  |  |  |
|  |  |  |  |  |  |  |  |  |
|  |  |  |  |  |  |  |  |  |
|  |  |  |  |  |  |  |  |  |
|  |  |  |  |  |  |  |  |  |
|  |  |  |  |  |  |  |  |  |
|  |  |  |  |  |  |  |  |  |
|  |  |  |  |  |  |  |  |  |
|  |  |  |  |  |  |  |  |  |
|  |  |  |  |  |  |  |  |  |

* Fill in environment type No.: 1. Urban ; 2. Rural; 3. Park and Bamboo; 4. Construction Site, Salvage Station, Tire factory; 5. Others

Name of Organization: Monitor: Reviewer:

**Table 5__**

**____________(** ***Ae. Albopictus*) Collection information**

Place： Province (Autonomous Region/Municipality) City(county) Township (street) Village (Neighborhood Committee)

| No. | Time | Place | Habitat | Longitude and Latitude | Quantity | | | Remark |
| --- | --- | --- | --- | --- | --- | --- | --- | --- |
|  |  |  |  |  | Adult mosquitoes | The larvael | Pupa |  |
|  |  |  |  |  |  |  |  |  |
|  |  |  |  |  |  |  |  |  |
|  |  |  |  |  |  |  |  |  |
|  |  |  |  |  |  |  |  |  |
|  |  |  |  |  |  |  |  |  |
|  |  |  |  |  |  |  |  |  |
|  |  |  |  |  |  |  |  |  |
|  |  |  |  |  |  |  |  |  |
|  |  |  |  |  |  |  |  |  |
|  |  |  |  |  |  |  |  |  |
|  |  |  |  |  |  |  |  |  |
|  |  |  |  |  |  |  |  |  |

Name of Organization: Collector: Method:

**Table 6______**

**Vector resistance (Virulence regression line)**

Province (Autonomous Region/Municipality) City(county) Township (street)

| Insect: ___________________  Drug: ___________________  Tester: ___________________  Insect State: ______  Age: ___________  Temperature: _ __℃;  Relative Humidity___% | | | Insect Source: ___________________________  Longitude: ____________  Latitude: _____________  Drug name: ___________________________  Date:_______Year___Month__Date to___month_ date  Temperature:_______℃;  Relative Humidity:_____% | | | | |
| --- | --- | --- | --- | --- | --- | --- | --- |
| Concentration/dose | Take 1 | | Take 2 | | Take 3 | | Total |
|  | death | Total death | death | Total death | death | Total death | death/total death |
| Control |  |  |  |  |  |  | / |
|  |  |  |  |  |  |  | / |
|  |  |  |  |  |  |  | / |
|  |  |  |  |  |  |  | / |
|  |  |  |  |  |  |  | / |
|  |  |  |  |  |  |  | / |
|  |  |  |  |  |  |  | / |
| Number of insects: ______________ Virulence regression line: ______________  χ^2^: _________________  Slope B value (95% confidence Interval) :______________________  LC_50_/LD_50_：____________________ 95% CI: __________________________  LC_95_/LD_95_：____________________95% CI: __________________________ | | | | | | | |
| * LC50 (mg/L) for mosquito larvae | | | | | | | |
| Name of Organization: Monitor: Reviewer: | | | | | | | |

**Table 7**

**Vector resistance (Diagnostic dose)**

Province (Autonomous Region/Municipality) City(county) Township (street)

| Insect: ___________________  Drug: ___________________  Tester: ___________________  Insect State: ______  Age: ___________  Temperature: _ __℃;  Relative Humidity___% | | | Insect Source: ___________________________  Longitude: ____________  Latitude: _____________  Drug name: ___________________________  Date:_______Year___Month__Date to___month_ date  Temperature:_______℃;  Relative Humidity:_____% | | | | |
| --- | --- | --- | --- | --- | --- | --- | --- |
| Repetition | Control | | | A Pesticides | | B Pesticides | |
|  |  |  |  | Dose/  Time | | Dose/  Time | |
|  | Death | Total death | | Death | Total death | Death | Total death |
| I |  |  | |  |  |  |  |
| II |  |  | |  |  |  |  |
| III |  |  | |  |  |  |  |
| IV |  |  | |  |  |  |  |
| V |  |  | |  |  |  |  |
| VI |  |  | |  |  |  |  |
| Total |  |  | |  |  |  |  |
| *This table is applicable to the determination of adult mosquito resistance by diagnostic dose. | | | | | | | |
| \| Name of Organization: Monitor: Reviewer: \| \| --- \| | | | | | | | |
